# Magnesium Transporter SLC41A1 Links Magnesium Homeostasis to NMDA Receptor-Related Synaptic Dysfunction: A Transdiagnostic Therapeutic Target for Neuropsychiatric Disorders

**DOI:** 10.3390/biomedicines14030610

**Published:** 2026-03-09

**Authors:** Xinru Chen, Wenhao Deng, Xinrui Chen, Yang Yu

**Affiliations:** 1 Guangdong Engineering Technology Research Center for Translational Medicine of Mental Disorders, Guangzhou 510370, China; dsxinsky@163.com (X.C.); vanhol_d@163.com (W.D.); chenxinrui20@163.com (X.C.); 2 The Affiliated Brain Hospital, Guangzhou Medical University, Guangzhou 510370, China; 3 Department of Clinical Psychology, The Affiliated Brain Hospital, Guangzhou Medical University, Guangzhou 510370, China; 4 Institute of Psycho-Neuroscience, The Affiliated Brain Hospital, Guangzhou Medical University, Guangzhou 510370, China

**Keywords:** neuropsychiatric disorders, SLC41A1, causal inference, multi-omics, magnesium homeostasis, NMDA receptor

## Abstract

**Background**: Neuropsychiatric disorders such as Alzheimer’s disease (AD), bipolar disorder (BD), and depression exhibit shared glutamatergic abnormalities, although their upstream molecular mechanisms remain poorly defined. Magnesium (Mg^2+^) serves as a key regulator of N-methyl-D-aspartate (NMDA) receptor function; however, the role of Mg^2+^ transporters, particularly SLC41A1, has not been systematically investigated. As NMDA receptor dysregulation contributes to emotional and cognitive impairments, elucidating Mg^2+^-NMDA signaling may enable the development of novel therapeutic strategies. **Methods**: We integrated Mendelian randomization, locus colocalization, human brain transcriptomics, functional enrichment, and co-expression analyses to determine whether SLC41A1 functions as a cross-disorder molecular driver. In addition, in vitro electrophysiological experiments using field potential recordings in hippocampal Schaffer-CA1 synapses were conducted to validate its functional role in NMDA receptor-mediated synaptic transmission. **Results**: Genetically elevated SLC41A1 expression increased the risk of AD, BD, depression, and alcohol dependence, with strong colocalization analyses supporting shared causal variants. Transcriptomic profiling revealed SLC41A1 upregulation in AD and BD, with enrichment in magnesium transport, mitochondrial function, and synaptic signaling pathways. Co-expression networks across GTEx brain regions demonstrated strong correlations with NMDA-related genes (e.g., *GRINA*, *CAMK2G*, *GRIN2C*). Under NMDAR-selective recording conditions, both imipramine treatment and SLC41A1 knockdown significantly reduced NMDAR-mediated fEPSP amplitudes, supporting a role for SLC41A1 in regulating NMDA receptor-dependent synaptic responses. **Conclusions**: This study identifies SLC41A1 as a magnesium-centered, transdiagnostic therapeutic target that links Mg^2+^ homeostasis to NMDA-dependent synaptic dysfunction. These findings provide a mechanistic foundation for developing SLC41A1-modulating or magnesium-based therapeutic approaches for mood and cognitive disorders.

## 1. Introduction

Neuropsychiatric disorders, including Alzheimer’s disease (AD), Parkinson’s disease (PD), depression, and bipolar disorder (BD), are major contributors to disability, premature mortality, and socioeconomic burden worldwide [1,2,3]. Although their clinical and epidemiological characteristics are well defined, the underlying molecular mechanisms remain poorly understood. The inherent complexity and heterogeneity of these disorders, together with the limitations of observational research, hinder the identification of causal biological pathways and therapeutic targets [4,5]. These challenges highlight the need for integrative analytical frameworks that leverage genetic instruments in combination with brain multi-omics data to infer causal mechanisms across disorders, rather than within isolated disease contexts.

A growing body of evidence implicates glutamatergic signaling, particularly N-methyl-D-aspartate (NMDA) receptor-dependent transmission, in the pathophysiology of neuropsychiatric conditions. Both acute and chronic stress elevate extracellular glutamate concentrations in the prefrontal cortex and hippocampus, resulting in NMDA receptor-mediated excitotoxicity and synaptic dysfunction in animal models of depression. On the other hand, pharmacological blockade of NMDA receptors by ketamine induces rapid and robust antidepressant effects, emphasizing the pivotal role of NMDA receptor dysregulation in mood disorders [6]. Beyond depression, aberrant NMDA receptor function has been implicated in schizophrenia, AD, and other neuropsychiatric conditions [7,8].

Magnesium (Mg^2+^) homeostasis serves as a critical upstream regulator of NMDA receptor activity. Acting as a voltage-dependent physiological blocker of the NMDA receptor channel, Mg^2+^ modulates synaptic plasticity, neuronal excitability, and susceptibility to excitotoxic damage [9,10]. Furthermore, Mg^2+^ contributes to mitochondrial function, oxidative stress regulation, and inflammatory signaling processes intricately linked to the pathophysiology of neuropsychiatric disorders [11]. Epidemiological and clinical evidence associates altered magnesium status with the risk and progression of AD, depression, BD, and PD. Several studies suggest that magnesium supplementation may ameliorate depressive symptoms in specific populations [12,13,14]. However, most investigations have concentrated on systemic magnesium levels, leaving the identity and function of Mg^2+^ transporters that maintain brain neuronal Mg^2+^ homeostasis largely unexplored. Collectively, these findings indicate that Mg^2+^-NMDA signaling may constitute a shared, upstream mechanism across mood and cognitive disorders.

Solute carrier family 41 member 1 (SLC41A1) is the most extensively characterized member of the SLC41 family, functioning as a Na^+^/Mg^2+^ exchanger that mediates Mg^2+^ efflux and maintains intracellular Mg^2+^ homeostasis [15,16]. SLC41A1 is widely expressed across human tissues, including dopamine-innervated brain regions such as the striatum [16,17]. Genetic studies have linked the PARK16 locus, which contains SLC41A1, to PD susceptibility, while functional analyses of the p.A350V variant demonstrate gain-of-function Mg^2+^ efflux and altered cyclic AMP-dependent regulation, contributing to dopaminergic neuron vulnerability [18]. In toxin-based PD models, SLC41A1 and other magnesium transporters are dynamically regulated and respond to magnesium supplementation [17,19]. Nevertheless, these findings have primarily been confined to PD and experimental neurodegeneration, leaving it uncertain whether SLC41A1 contributes to a broader spectrum of neuropsychiatric conditions or how it is integrated within human brain transcriptional and regulatory networks [20,21].

In this study, we tested the hypothesis that SLC41A1 functions as a shared molecular driver linking Mg^2+^ homeostasis to NMDA receptor-associated synaptic dysfunction across neuropsychiatric disorders. To address this, we combined expression-based Mendelian randomization with locus colocalization, human brain transcriptomics, co-expression and regulatory network analyses, and electrophysiological validation. Specifically, we (i) assessed the causal effect of SLC41A1 expression on the risk of 13 neuropsychiatric disorders and (ii) delineated SLC41A1-centered molecular pathways within the human brain, examining their functional consequences at Schaffer-CA1 synapses. By integrating evidence across these complementary analytical levels of analysis, we sought to determine whether SLC41A1 represents a magnesium-centered, transdiagnostic susceptibility gene and to identify its downstream pathways as potential targets for therapeutic intervention in neuropsychiatric diseases.

## 2. Materials and Methods

### 2.1. Study Design and Overview

We conducted an integrative analysis employing multiple complementary: (1) two-sample Mendelian randomization (MR) with locus colocalization, (2) independent transcriptomic profiling, (3) regulatory network inference, (4) brain region-specific co-expression analyses, and (5) electrophysiological recordings. The primary objective was to estimate the causal effect of SLC41A1 expression on neuropsychiatric disease risk and to triangulate convergent evidence from genetic, molecular, transcriptomic, and physiological datasets. All analyses were performed according to prespecified protocols and conformed to the STROBE-MR and ARRIVE guidelines.

### 2.2. Data Sources

Expression quantitative trait locus (eQTL) summary statistics for SLC41A1 were obtained from eQTLGen (whole blood; *n* = 31,684) [22]. When multiple probes or single-nucleotide polymorphisms (SNPs) are mapped to the locus, cis-eQTLs within ±1 Mb of the transcription start site were retained.

Genome-wide association study (GWAS) summary statistics were compiled for thirteen neuropsychiatric disorders, including AD [23], PD [23], depression [24], BD [25], schizophrenia [26], anxiety disorders [27], posttraumatic stress disorder (PTSD) [23], obsessive–compulsive disorder (OCD) [28], attention-deficit hyperactivity disorder (ADHD) [29], autism spectrum disorder (ASD) [30], anorexia nervosa [31], alcohol dependence [23], and smoking behavior [32]. Data harmonization ensured genome build consistency, corrected strand alignment, and eliminated duplicate or mismatched variants.

Case–control transcriptomic datasets for AD (GSE122063), BD (GSE46416), and depression (GSE35978) were retrieved from the Gene Expression Omnibus (GEO) [33]. These three outcomes were prioritized for transcriptomic analyses based on prior evidence of MR associations and strong colocalization with SLC41A1 expression. Regulatory information was obtained from miRTarBase (experimentally validated miRNA–target interactions) and ENCODE ChIP-seq (transcription factor-binding sites) [34,35]. Brain region-specific RNA-seq data (TPM-normalized) were acquired from GTEx v10 for the amygdala, frontal cortex (BA9), and hippocampus [36].

### 2.3. Mendelian Randomization Analysis

#### 2.3.1. Instrument Selection and Harmonization

Instrumental variables for SLC41A1 expression were selected from the eQTLGen consortium. Disease-specific significance thresholds were applied to ensure sufficient instrument strength while minimizing potential bias: *p* <  5 × 10^−6^ for anxiety disorder [37], depression, anorexia nervosa, and smoking; *p* <  5 × 10^−7^ for schizophrenia; and *p* <  5 × 10^−8^ for AD, PD, BD, PTSD, ASD, OCD, ADHD, and alcohol dependence [38]. Independent variants were identified through linkage disequilibrium (LD) clumping (*r*^2^ < 0.001; 10 Mb window) using the ‘ieugwasr’ R package [37]. Variants with a minor allele frequency (MAF) ≥0.01 were retained.

Instruments demonstrating weak strength (F ≤ 10) or ambiguous strand alignment were excluded. Exposure and outcome datasets were harmonized to ensure consistent effect allele alignment, and variants with significant genome-wide associations for outcomes (*p* < 5 × 10^−8^) were removed. Potential pleiotropic effects were assessed using LDlink phenotype scans; SNPs highly correlated (*r*^2^ > 0.8) with other risk loci were also excluded. Detailed characteristics of the instrumental variables are provided in Supplementary Table S1.

#### 2.3.2. Causal Estimation and Sensitivity Analyses

Primary causal estimates were calculated using the inverse-variance weighted (IVW) method with a multiplicative random-effects model, implemented in the ‘TwoSampleMR’ R package (Version 0.5.10) [39]. To enhance the robustness of causal inference, complementary analyses were performed using MR-Egger regression, weighted median, simple-mode, and weighted-mode estimators. Cochran’s Q statistics were used to assess the MR-Egger intercept and the MR-PRESSO global to test heterogeneity and horizontal pleiotropy. Leave-one-out sensitivity analyses were conducted to assess the influence of individual variants on overall estimates. Multiple comparisons across thirteen outcomes were corrected using Bonferroni adjustment (*α* = 0.05/13).

#### 2.3.3. Colocalization Analysis

Locus-specific colocalization analyses were conducted using the ‘coloc’ R package [40]. Outcomes were prespecified for colocalization if they satisfied the MR-based inclusion criteria, defined as either Bonferroni-corrected significance in the IVW analysis or a nominal IVW *p* < 0.05. Variants located within ±1 Mb of the SLC41A1 transcription start site were harmonized, and strong colocalization evidence was defined as a posterior probability for Hypothesis 4 (PPH4) > 0.8.

### 2.4. Transcriptomic Analyses

#### 2.4.1. Preprocessing and Differential Expression

The raw data were downloaded from GEO and preprocessed using standard procedures, including background correction, log_2_ transformation, and quantile normalization. Differential expression analyses were performed with the ‘limma’ R package [41]. Genes with a false discovery rate (FDR)-adjusted *p* value < 0.05 were considered statistically significant. Expression differences in SLC41A1 between cases and control groups were explicitly evaluated within each cohort.

#### 2.4.2. Functional Enrichment Analysis

Gene Ontology (GO) and Kyoto Encyclopedia of Genes and Genomes (KEGG) pathway analyses were conducted using the ‘clusterProfiler’ R package [42]. Single-sample Gene Set Enrichment Analysis (ssGSEA) was applied to RNA-seq data from AD, BD, and depression patients to compute normalized ssGSEA scores for each sample. Group differences in these scores were evaluated using linear modeling with FDR adjustment to control for multiple comparisons.

#### 2.4.3. Correlation Analysis Between SLC41A1 and Disease-Associated Genes

Disease-associated genes for AD, BD, and depression were retrieved from the GeneCards database [43], along with their GeneCards relevance scores. For each disorder, differentially expressed genes (DEGs) were identified from the respective case–control RNA-seq datasets (adjusted *p* < 0.05), resulting in sets of disease-specific DEGs. Normalized log_2_ expression values from the RNA-seq data were then used to calculate Pearson correlation coefficients between SLC41A1 and each candidate gene within each disorder-specific cohort, using the ‘cor’ R package. Genes exhibiting strong co-expression with SLC41A1 (|*r*| > 0.8 and *p* < 0.05) were defined as highly correlated, and the top 20 correlated genes per disorder were visualized as clustered correlation heatmaps.

#### 2.4.4. Co-Expression with NMDA Receptor Genes

The GTEx v10 dataset was utilized to examine the co-expression patterns of SLC41A1 with 15 NMDA receptor-related genes (e.g., *GRIN1*, *GRIN2A*, *GRIN2B*, *CAMK2G*) across brain tissues. Pearson correlation coefficients were calculated to assess co-expression between SLC41A1 and these target genes in three brain regions—the amygdala, frontal cortex (BA9), and hippocampus—using RNA-seq TPM data. Statistical significance was defined as *p* < 0.05.

#### 2.4.5. Regulatory Network Inference

Regulatory networks of SLC41A1 were inferred by integrating experimentally validated miRNA–target interactions from miRTarBase and transcription factor-binding information from ENCODE. These interactions were used to construct both miRNA-SLC41A1 and TF-SLC41A1 regulatory networks. Comprehensive methodological details and the full network analyses are presented in the Supplementary Materials.

### 2.5. Electrophysiological Recordings

The experimental protocol was reviewed and approved by the Institutional Animal Care and Use Committee of Guangzhou Medical University (Approval No.: GY2022-214). All animal experiments were performed following the Chinese Guidelines for the Care and Use of Laboratory Animals. Adult male C57BL/6J mice (6–8 weeks, 20–25 g, from Guangdong Medical Laboratory Animal Center, Guangzhou, China) were housed at 4–5 per cage under a 12 h light/dark cycle (lights on from 7:00 a.m. to 7:00 p.m.), with ad libitum access to food and water.

Acute hippocampal slices were prepared and maintained in oxygenated artificial cerebrospinal fluid (ACSF). Hippocampal slices (250–300 μm) were incubated in ACSF containing (in mM): 120.0 NaCl, 2.5 KCl, 1.0 MgCl_2_, 25.0 NaHCO_3_, 1.25 NaH_2_PO_4_, 2.0 CaCl_2_, 25.0 glucose, and 1.0 ascorbic acid. All recordings were performed in the CA1 region of the hippocampus. Extracellular field excitatory postsynaptic potentials (fEPSPs) were recorded using 3–4 MΩ ACSF-filled electrodes and a MultiClamp 700B amplifier (Molecular Devices, Sunnyvale, CA, USA). Responses were elicited by electrical stimulation of the Schaffer collateral pathway at a frequency of 0.05 Hz using single 0.1 ms pulses delivered through a concentric electrode.

The initial amplitudes of the fEPSP were measured, expressed as the baseline level. To isolate NMDAR-mediated fEPSP, bath application of DNQX (AMPAR antagonist; 10 μM, Sigma-Aldrich, St. Louis, MO, USA) in Mg^2+^-free ACSF was carried out. To evaluate the functional role of SLC41A1 in NMDA receptor-mediated synaptic responses, we employed two complementary approaches: acute pharmacological inhibition using imipramine (100 μM, Sigma-Aldrich, St. Louis, MO, USA) [15] and specific genetic knockdown through AAV-mediated shRNA delivery targeting SLC41A1 (rAAV-hSyn-mCherry-5′miR-30a-shRNA (SLC41A1), shRNA sequence: GAGGTCTCATCCTGGACAAGA, titer: 5.5 × 10^12^ v.g./mL). A scramble virus (rAAV-hSyn-mCherry-5′miR-30a-shRNA (scramble), sequence: CCTAAGGTTAAGTCGCCCTCG, titer: 5.0 × 10^12^ v.g./mL) was used as a scramble control.

Mice were deeply anesthetized (Pentobarbital NEMBUTAL, 100 mg/kg, intraperitoneal injection) and head-fixed in a stereotaxic instrument (Stoelting Instruments, Wood Dale, IL, USA). Virus was injected into the target brain regions (CA1 regions: AP, −1.80 mm; ML, ± 1.50 mm; DV, −1.61 mm) using a glass micropipette connected to a pressure microinjector (Microinjection Syringe Pump, WPI, Sarasota, FL, USA) at a speed of 50 nL/min. A total volume of 200 nL was delivered per side. The micropipette was left in place for 10 min to allow the injectant to diffuse adequately. After surgery, mice recovered from anesthesia on a heat pad.

Three weeks after viral injection, injection accuracy was verified by mCherry fluorescence in CA1. Knockdown efficiency was assessed by measuring SLC41A1 protein levels in hippocampal tissue. Synaptic function was evaluated by recording fEPSPs in hippocampal slices under NMDAR-selective conditions (Mg^2+^-free ACSF supplemented with DNQX).

For quantitative analysis, we calculated the ratio of the NMDAR-mediated fEPSP amplitude to the baseline fEPSP amplitude. The experiment included two groups: a control group and an imipramine-treated group. In total, 5 slices per group (one slice per mouse; 5 mice per group) were included in the analysis. Hippocampal slices from different animals were randomly allocated to the control or imipramine-treated conditions using a computer-generated randomization list. In the viral knockdown experiment, two groups were analyzed: a scramble control group (10 slices from 3 mice) and an SLC41A1 shRNA group (11 slices from 3 mice). Sample size was based on our previous experience with similar electrophysiological experiments and is comparable to that used in other mechanistic physiology studies, rather than on a formal a priori power calculation.

Data were excluded a priori if they showed unstable baselines (>20% change in fEPSP amplitude during the 10 min baseline period), poor signal-to-noise ratios, or loss of synaptic responses during drug application. Recordings from control and imipramine-treated slices were interleaved on the same experimental day to minimize potential confounding by time-dependent changes in slice quality or recording stability. All electrophysiological recordings and offline data analyses were performed by an experimenter who was blinded to group allocation.

Graphical representations and statistical analyses were generated using GraphPad Prism version 10, with data presented as mean ± standard error of the mean (SEM). Comparisons between the two groups were performed using Student’s two-tailed independent samples *t*-test, with * *p* < 0.05 considered statistically significant.

## 3. Results

### 3.1. Causal Effects of SLC41A1 on Neuropsychiatric Disorders

The primary MR analysis conducted using the IVW methods identified four neuropsychiatric disorders in which genetically proxied SLC41A1 expression was significantly associated with increased disease risk, surpassing the Bonferroni-corrected significance threshold (*α* = 0.0038). These disorders included AD (odds ratio [OR] = 1.20, 95% CI 1.09–1.32, *p* = 1.33 × 10^−4^), BD (OR = 1.14, 95% CI 1.06–1.22, *p* = 5.59 × 10^−4^), depression (OR = 1.11, 95% CI 1.07–1.15, *p* = 7.65 × 10^−8^), and alcohol dependence (OR = 1.18, 95% CI 1.08–1.29, *p* = 2.90 × 10^−4^).

**Figure 1:**
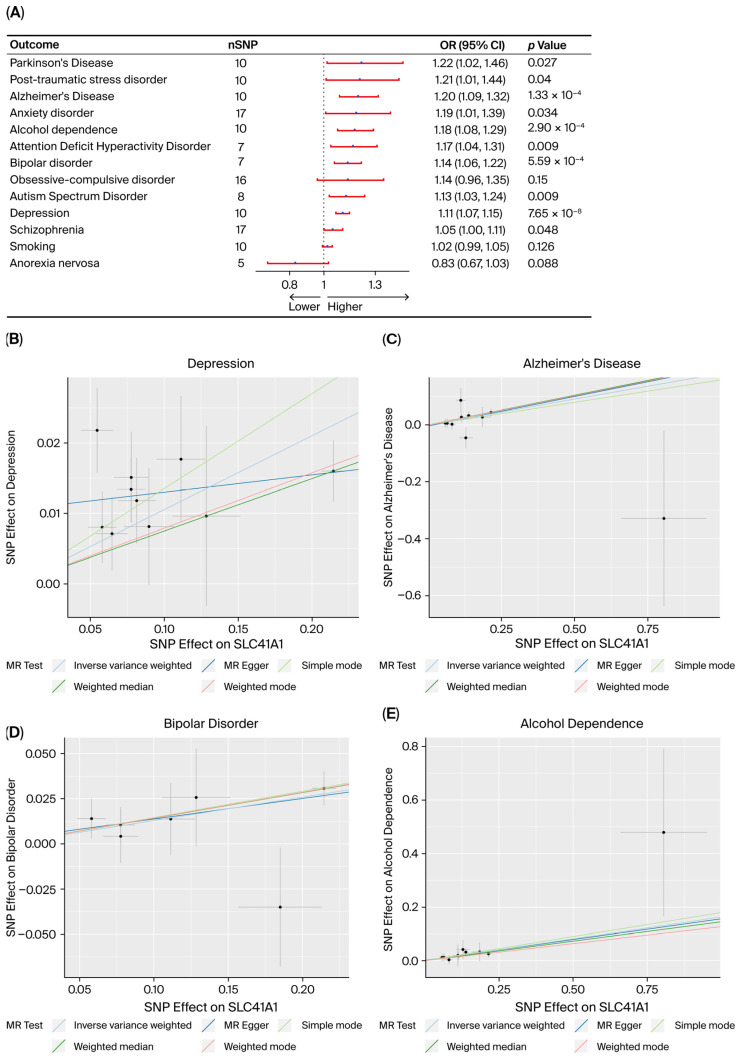
Mendelian Randomization (MR) analysis of SLC41A1 and thirteen neuropsychiatric disorders. (**A**) Blue dots represent the estimated odds ratios (ORs), and red horizontal lines indicate the corresponding 95% confidence intervals (CIs). The vertical black dotted line denotes the null value (OR = 1). The number of instrumental single-nucleotide polymorphisms (nSNP) and the corresponding *p*-value for each disorder are also indicated. (**B**–**E**) MR test results for four representative disorders: (**B**) Depression, (**C**) Alzheimer’s disease, (**D**) Bipolar disorder, and (**E**) Alcohol dependence. Each panel illustrates the relationship between the effect of individual SNPs on SLC41A1 expression (x-axis) and their effect on disease risk (y-axis). Each black dot represents an individual instrumental SNP. The horizontal and vertical gray lines around each dot indicate the standard errors (SEs) for the SNP-exposure and SNP-outcome associations, respectively. The colored diagonal lines represent the causal effect estimates derived from five different MR methods: inverse-variance weighted, MR Egger, simple mode, weighted median, and weighted mode.

Six additional outcomes showed nominal associations (*p* < 0.05), but these associations did not remain significant after correction for multiple testing corrections: anxiety disorder, schizophrenia, PD, ADHD, ASD, and PTSD. No significant IVW associations were detected for OCD, anorexia nervosa, or smoking behavior. A summary forest plot for the IVW results across all outcomes is presented in Figure 1.

### 3.2. Robustness and Sensitivity Analyses

Robustness was evaluated using multiple complementary estimators. The weighted-median estimator remained statistically significant for the four outcomes that surpassed the significant after Bonferroni-corrected threshold—depression, AD, BD, and alcohol dependence (all *p* < 0.05). The weighted-mode estimator confirmed the associations with depression, AD, and BD but did not replicate the association with alcohol dependence. MR-Egger slope estimates were nonsignificant across all four disorders, indicating that horizontal pleiotropy was unlikely to represent a major source of bias. Among the outcomes showing nominal associations, both PD and ADHD demonstrated consistent results across the weighted-median and weighted-mode estimators. ASD exhibited borderline significance with the weighted-median estimator (*p* ≈ 0.054), whereas no supporting evidence was observed for anxiety disorder, PTSD, and schizophrenia. Full results from the five MR estimators for each outcome are provided in Supplementary Table S2.

Cochran’s Q statistics were used to evaluate heterogeneity across the instrument-specific causal estimates. No significant heterogeneity was observed for most outcomes (all *p* > 0.05). However, marginal heterogeneity was noted for PD (Q = 16.854, df = 9, *p* = 0.051), suggesting a minor inconsistency among SNP-specific causal estimates. To further confirm the robustness of the findings, leave-one-out sensitivity analyses were performed for all thirteen outcomes. For each outcome, the effect estimates remained stable across all iterations, indicating that no single SNP exerted a disproportionate influence on the overall causal estimate. These findings collectively support the reliability of the observed associations. A complete summary of the leave-one-out results for all outcomes is presented in Supplementary Figure S1.

Horizontal pleiotropy was evaluated using MR-Egger intercepts and MR-PRESSO diagnostics. MR-Egger intercepts were nonsignificant for nearly all outcomes, except for depression, where the intercept differed significantly from zero (*p* = 0.024), suggesting possible pleiotropy for this outcome. Additionally, MR-PRESSO did not identify evidence of significant pleiotropy for any outcome (all global *p* > 0.05). A summary of heterogeneity and horizontal pleiotropy diagnostics is provided in Table 1.

### 3.3. Colocalization of SLC41A1 Expression and Neuropsychiatric Disorders

**Figure 2:**
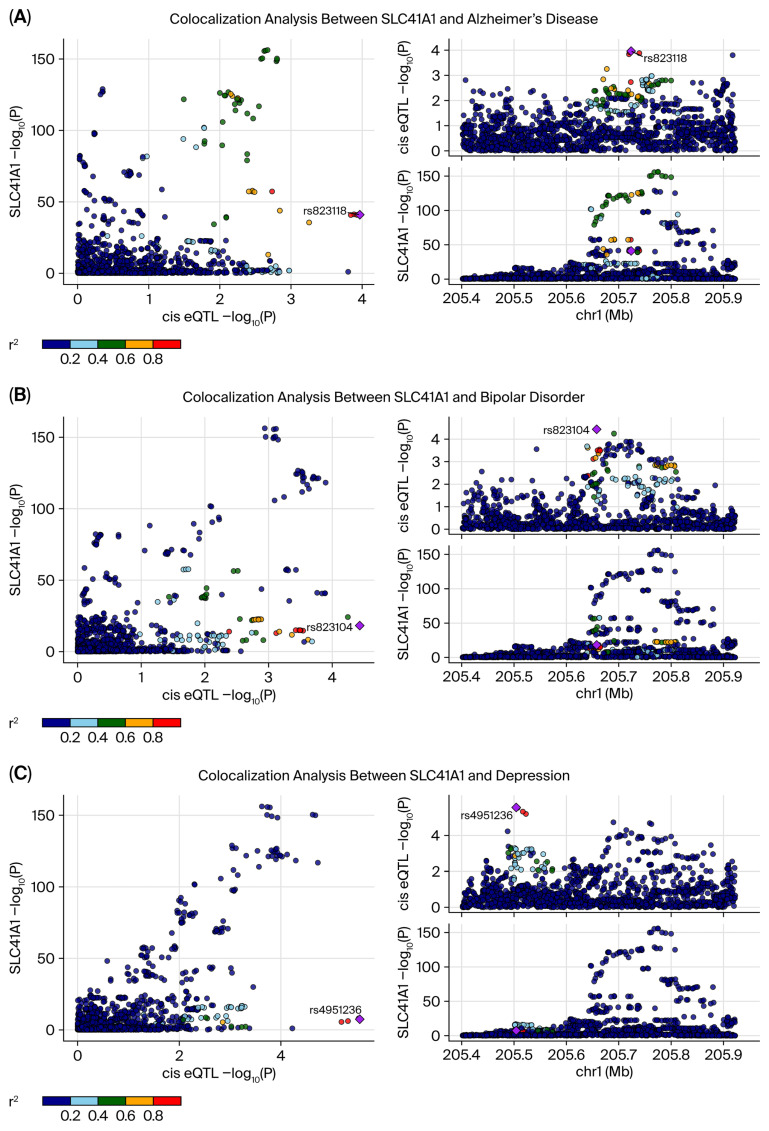
Co-localization analysis between SLC41A1 and neuropsychiatric disorders. Co-localization analyses were conducted to assess the relationship between SLC41A1 expression and genetic variants associated with three neuropsychiatric disorders: (**A**) Alzheimer’s disease, (**B**) Bipolar disorder, and (**C**) depression. Each panel depicts the association between SLC41A1 expression (*y*-axis) and the corresponding cis-eQTL/SNP (*x*-axis), with color indicating the strength of the correlation (*r*^2^). In each panel, the right lower corner zooms in on the SLC41A1 genomic region (chromosome 1), highlighting the relevant SNPs. These findings demonstrate a shared genetic mechanism involving SLC41A1 expression across these neuropsychiatric conditions, with specific SNPs consistently showing strong associations.

Colocalization analysis was performed for ten outcomes that satisfied the MR inclusion criteria, comprising four Bonferroni-significant and six nominally significant disorders. Strong evidence of colocalization was detected for depression (PPH4 = 0.973), BD (PPH4 = 0.940), and AD (PPH4 = 0.881), indicating substantial shared genetic signals between SLC41A1 expression and these disorders. Moderate colocalization was observed for PD (PPH4 = 0.613), suggesting less pronounced but still notable genetic degree of overlap. For the remaining outcomes, the PPH4 values were <0.5, indicating limited or no significant genetic overlap. Representative colocalization plots for AD, BD, and depression are shown in Figure 2. The complete posterior probability vectors (PPH0–PPH4) for all ten outcomes are provided in Supplementary Table S3.

### 3.4. Differential Expression and Functional Enrichment Analysis of SLC41A1 in Neuropsychiatric Disorders

Transcriptomic profiling was conducted only for AD, BD, and depression, for which MR and colocalization analyses implicated SLC41A1.

#### 3.4.1. Differential Expression of SLC41A1 in Case–Control Transcriptomes

**Figure 3:**
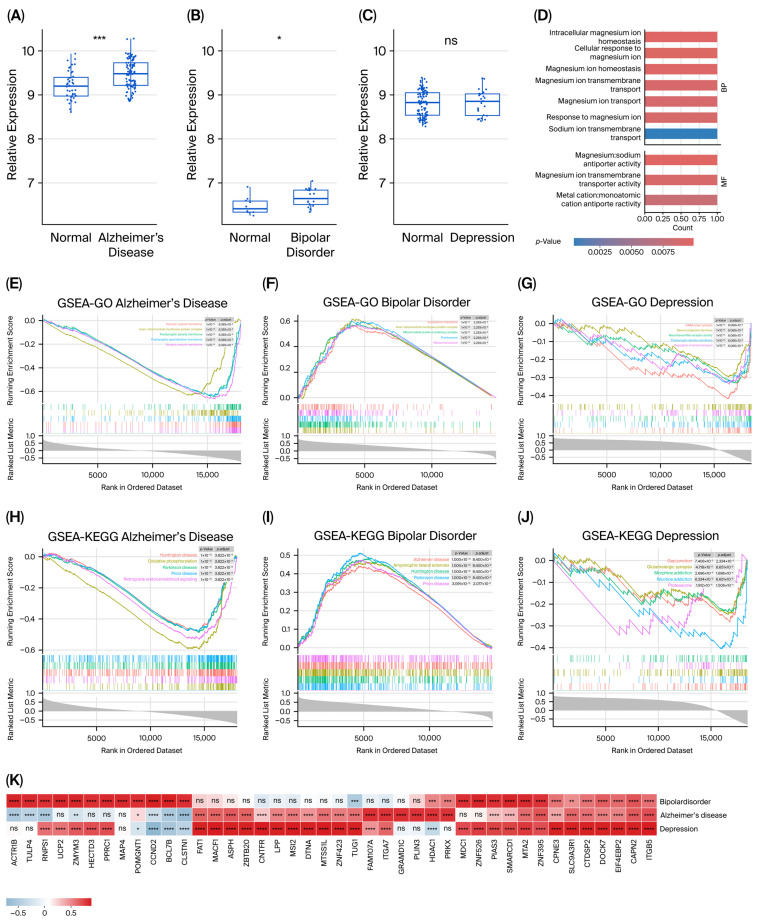
Transcriptomic analysis of SLC41A1 expression and associated pathways across neuropsychiatric disorders. (**A**–**C**) Differential expression of SLC41A1 in case–control transcriptomes. (**A**) Alzheimer’s disease, (**B**) Bipolar disorder, and (**C**) depression. The box plots show the relative SLC41A1 expression in normal vs. disease groups, with statistical significance indicated for Alzheimer’s Disease and Bipolar Disorder. (**D**) Gene Ontology (GO) analysis of SLC41A1-associated differentially expressed genes (DEGs), identifying 23 significant pathways. The top 10 pathways with the most significant enrichment are shown, including pathways related to magnesium ion transport and cellular response to magnesium ion. (**E**–**G**) ssGSEA-GO analysis of SLC41A1 expression in Alzheimer’s disease (**E**), Bipolar disorder (**F**), and depression (**G**). Each plot shows enrichment scores for various GO terms, highlighting cellular processes and functions enriched in the SLC41A1 expression data. (**H**–**J**) ssGSEA-KEGG analysis of SLC41A1 expression in Alzheimer’s disease (**H**), Bipolar disorder (**I**), and depression (**J**). The plots show enrichment scores for KEGG pathways, with notable pathways related to neurodegenerative diseases, synaptic function, and addiction. (**K**) The heatmap illustrates the correlation between SLC41A1 expression and the top 20 differentially expressed genes in Alzheimer’s disease, Bipolar disorder, and depression. Statistical significance is defined as: ns: *p* > 0.05; *: *p* < 0.05; **: *p* < 0.005; ***: *p* < 0.001; ****: *p* < 0.0001.

Differential expressions of SLC41A1 were examined in patients with AD, BD, and depression. The analysis revealed a significant upregulation of SLC41A1 expression in AD (*p* < 0.001) and BD (*p* < 0.05), whereas no significant differential expression of SLC41A1 was observed in patients with depression (*p* > 0.05). Representative box plots depicting the differential expression of SLC41A1 between case and control groups for each disorder are presented in Figure 3A–C.

#### 3.4.2. Functional Enrichment

GO analysis of SLC41A1 identified 23 significant pathways (*p* < 0.05) associated with SLC41A1. The most enriched terms included sodium antiporter activity (*p* = 9.7 × 10^−4^), intracellular magnesium ion homeostasis (*p* = 1.8 × 10^−3^), the cellular response to magnesium ions (*p* = 1.8 × 10^−3^), and magnesium ion transmembrane transport (*p* = 1.8 × 10^−3^). Representative enriched GO terms are shown in Figure 3D, and the complete list of all enriched pathways is provided in Supplementary Table S4. No significant enrichment was detected in the KEGG pathways.

Single-sample gene set enrichment analysis (ssGSEA-GO) of SLC41A1 expression revealed significant enrichment in key pathways related to synaptic transmission and mitochondrial function across the three neuropsychiatric disorders. In AD, the most enriched pathways included synaptic vesicle membrane (*p* = 8.4 × 10^−9^) and inner mitochondrial membrane protein complex (*p* = 8.4 × 10^−9^). BD demonstrated strong enrichment in mitochondrial-related pathways, including inner mitochondrial membrane protein complex (*p* = 2.3 × 10^−8^) and mitochondrial protein-containing complex (*p* = 2.3 × 10^−8^). In depression, significant enrichment was primarily observed in synaptic transmission pathways, such as GABAergic synapse (*p* = 6.1 × 10^−8^) and neurotransmitter receptor activity (*p* = 6.1 × 10^−8^). Representative results for these enriched pathways are shown in Figure 3E–G.

ssGSEA-KEGG analysis revealed significant enrichment in pathways associated with neurodegenerative diseases and addiction. Both AD and BD samples exhibited significant enrichment in Parkinson’s disease (PD)-related pathways (*p* = 3.8 × 10^−9^ for AD, *p* = 8.4 × 10^−9^ for BD) and Huntington’s disease (*p* = 3.8 × 10^−9^ for AD, *p* = 8.4 × 10^−9^ for BD). In depression, significant enrichment was observed in addiction-related pathways, including morphine addiction (*p* = 1.7 × 10^−4^) and nicotine addiction (*p* = 6.7 × 10^−6^). Additional enrichment was found in gap junction (*p* = 2.3 × 10^−6^) and glutamatergic synapse (*p* = 6.7 × 10^−6^). The detailed enrichment results for these pathways are illustrated in Figure 3H–J.

#### 3.4.3. Correlation of SLC41A1 with Disease-Related Expression Profiles

To investigate the transcriptional role of SLC41A1 within disease-associated networks, correlations between SLC41A1 and differentially expressed genes (DEGs) were computed for AD, BD, and depression. For each disorder, the top 20 genes most strongly correlated with SLC41A1 were identified and visualized in a clustered heatmap (Figure 3K). Across all datasets, eleven genes, including *ITGB5*, *CAPN2*, *EIF4EBP*, and *DOCK7*, consistently exhibited strong positive correlations with SLC41A1. In addition to this three-way intersection, further pairwise overlaps were observed: thirteen genes shared by AD and depression, seven by BD and depression, and two by AD and BD. Overall, these results demonstrate that SLC41A1 is co-expressed with disease-related genes across AD, BD, and depression, forming a core network of shared partners and disease-specific co-expression patterns.

#### 3.4.4. Co-Expression with NMDA Receptor-Related Genes

**Figure 4:**
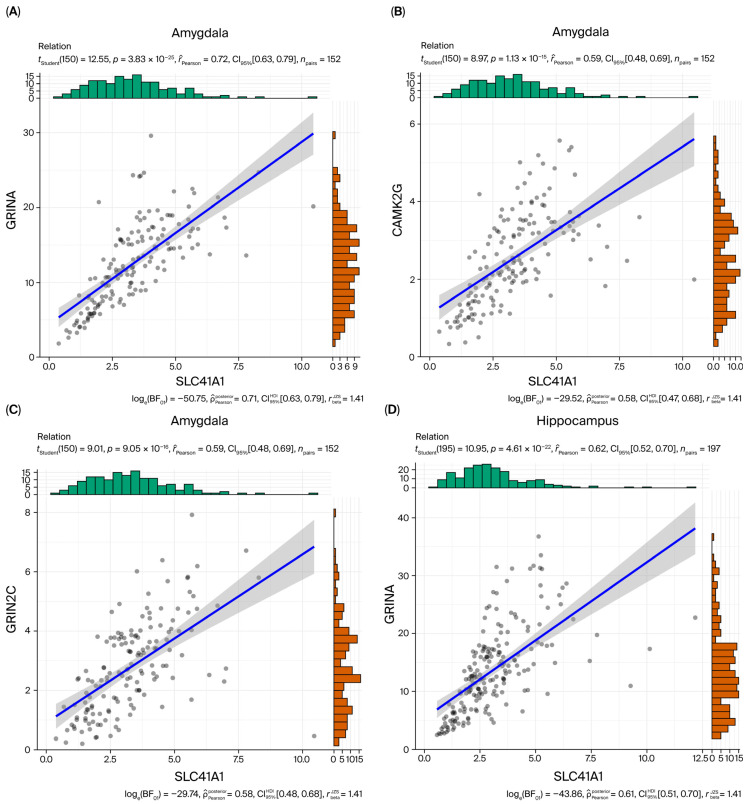

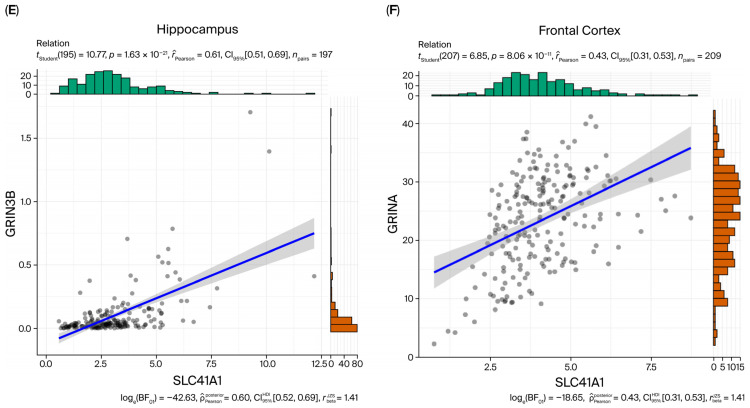
Co-expression analysis of SLC41A1 with NMDA receptor-related genes across brain regions (GTEx). Co-expression analysis was performed using Pearson correlation to examine the relationship between SLC41A1 and NMDA receptor-related genes (e.g., *GRINA*, *CAMK2G*, *GRIN2C*) across several brain regions, including the Amygdala (**A**–**C**), Hippocampus (**D**,**E**), and Frontal Cortex (**F**). Significant positive correlations were observed, particularly in the Amygdala and Hippocampus, suggesting a strong association between SLC41A1 expression and NMDA receptor-related genes in regions involved in emotional processing and memory. Each dot represents an individual sample. The blue solid lines indicate the fitted linear regression, and the surrounding gray shaded areas represent the 95% confidence intervals of the regression lines. The Pearson correlation coefficient (*r*) and corresponding *p* value are indicated in each panel.

In GTEx v10, SLC41A1 exhibited region-specific co-expression with NMDA receptor-related genes. The strongest correlation was observed in the amygdala, where SLC41A1 was highly associated with *GRINA* (*r* = 0.72, *p* = 3.8 × 10^−25^). *GRINA* also demonstrated consistent associations across other regions, with *r* = 0.62 in the hippocampus (*p* = 4.6 × 10^−22^) and *r* = 0.43 in the frontal cortex (*p* = 8.1 × 10^−11^). Additional strong associations were detected in the amygdala, with *CAMK2G* (*r* = 0.59, *p* = 1.1 × 10^−15^) and *GRIN2C* (*r* = 0.59, *p* = 9.1 × 10^−16^). In the hippocampus, SLC41A1 was also significantly associated with *GRIN3B* (*r* = 0.61, *p* = 1.6 × 10^−21^). Representative scatter plots are shown in Figure 4.

#### 3.4.5. Identification of miRNA and Transcription Factor Regulatory Networks of SLC41A1

Twelve miRNAs and several transcription factors (TFs)—including *ZNF76*, *ZBTB11*, and *RFX1*—were identified as potential regulators of SLC41A1 using data from miRTarBase and ENCODE. These regulatory elements constitute the miRNA-SLC41A1 and TF-SLC41A1 interaction networks, delineating key molecular interactions governing SLC41A1 expression. The complete results of the network analyses, including construction methods and topological parameters, are presented in Supplementary Figure S2.

### 3.5. SLC41A1 Regulates NMDAR-Mediated Synaptic Transmission in the Schaffer Collateral–CA1 Pathway

The Schaffer collateral–CA1 synapse is a fundamental neural pathway in the hippocampus, serving as a primary model for studying glutamatergic transmission and synaptic plasticity. It involves the projection of axons from CA3 pyramidal neurons to CA1 pyramidal neurons, forming excitatory synapses that rely on glutamate as the neurotransmitter, which activates NMDA and AMPA receptors on CA1 pyramidal neurons to mediate excitatory transmission and plasticity.

During the Electrophysiological recordings, the initial amplitudes of the field excitatory postsynaptic potentials (fEPSPs) were measured, expressed as the baseline level. To isolate NMDAR-mediated fEPSP, bath application of DNQX (AMPAR antagonist; 10 μM) in Mg^2+^-free ACSF was carried out. To assess the functional contribution of SLC41A1 to NMDA receptor-mediated synaptic responses, hippocampal slices were perfused with the SLC41A1 inhibitor imipramine (100 μM).

**Figure 5:**
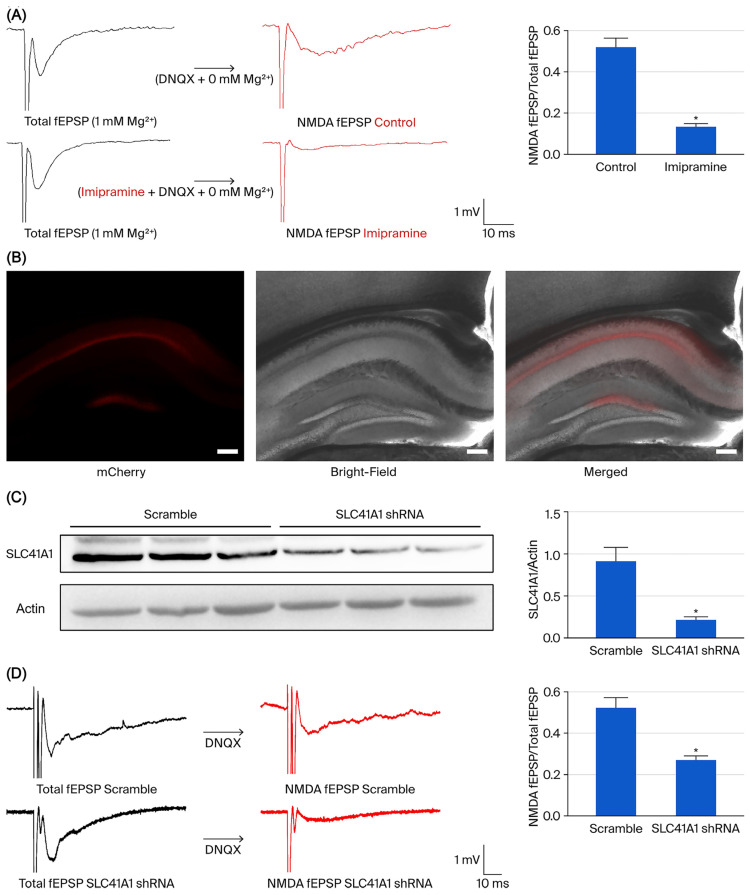
Electrophysiological recordings. (**A**) Electrophysiological recordings in the hippocampal Schaffer-CA1 region showed a decrease in NMDA receptor-mediated field potentials (NMDA fEPSP) in slices treated with 100 μM imipramine (an SLC41A1 inhibitor). The amplitude of NMDA fEPSP was significantly reduced in the imipramine group compared to controls (imipramine group, *n* = 5: 0.1370 ± 0.0116 vs. control group, *n* = 5: 0.5228 ± 0.0399; * *p* < 0.05, two-tailed Student’s *t*-test). Representative traces and stimulation/recording parameters are shown on the left, with quantified results on the right. (**B**) Representative images show location of injection accuracy for mCherry expression in CA1 region. Scale bar, 200 μm. (**C**) shRNA knockdown efficiency: SLC41A1 protein levels were quantified in hippocampal tissue of scramble and shRNA mice (scramble mice, *n* = 6: 0.9172 ± 0.1595 vs. shRNA mice, *n* = 6: 0.2178 ± 0.0335; * *p* < 0.05, two-tailed Student’s *t*-test). (**D**) SLC41A1 knockdown by shRNA significantly reduced the amplitude of NMDAR-mediated fEPSPs. The SLC41A1 shRNA group showed markedly lower fEPSP amplitudes than the scramble group (SLC41A1 shRNA group, *n* = 11: 0.2720 ± 0.0190 vs. scramble group, *n* = 10: 0.5244 ± 0.0477; * *p* < 0.05, two-tailed Student’s *t*-test). Representative traces and stimulation/recording parameters are shown on the left, with quantified results on the right.

Electrophysiological recordings from the hippocampal Schaffer-CA1 region demonstrated that treatment with 100 μM imipramine (a known SLC41A1 inhibitor) [15] significantly reduced the amplitude of NMDA receptor-mediated fEPSPs. The imipramine-treated group exhibited markedly lower fEPSP amplitudes (*n* = 5; 0.1370 ± 0.0116) compared to controls (*n* = 5; 0.5228 ± 0.0399, *p* < 0.05) (Figure 5A).

Three weeks after viral SLC41A1 shRNA injection, injection accuracy was verified by mCherry fluorescence in CA1 (Figure 5B). Knockdown efficiency was assessed by measuring SLC41A1 protein levels in hippocampal tissue (Figure 5C, Supplementary Figure S3).

Similarly, SLC41A1 knockdown by shRNA significantly reduced the amplitude of NMDAR-mediated fEPSPs. The SLC41A1 shRNA group showed markedly lower fEPSP amplitudes (*n* = 11, 0.2720 ± 0.0190) than the scramble group (*n* = 10, 0.5244 ± 0.0477; *p* < 0.05) (Figure 5D). These findings support an association between SLC41A1 perturbation and reduced NMDAR-mediated synaptic responses in the Schaffer collateral–CA1 pathway.

## 4. Discussion

This study provides multi-level evidence that SLC41A1, a critical magnesium transporter involved in neuronal excitability and synaptic plasticity, plays a significant role in the pathogenesis of several neuropsychiatric disorders, including AD, BD, depression, and alcohol dependence. Using MR combined with colocalization analysis, we established a robust causal relationship between SLC41A1 expression and the risk of these conditions, underscoring its contribution to shared genetic susceptibility. Transcriptomic analyses revealed consistent up-regulation of SLC41A1 in AD and BD patients, but no significant change in depression. Nevertheless, the strong co-expression between SLC41A1 and NMDA receptor-related genes supports a role in glutamatergic signaling. Moreover, pharmacological inhibition of SLC41A1 reduced NMDA receptor-mediated fEPSPs, providing direct functional evidence for the SLC41A1-NMDA pathway’s role in neuroplasticity. Collectively these findings position SLC41A1 as a potentially targetable molecule and establish a foundation for future studies aimed at modulating SLC41A1-dependent NMDA signaling in neuropsychiatric diseases.

At the genetic level, MR and colocalization analyses support a causal role for SLC41A1 expression in the risk for neuropsychiatric disorders, with locus-specific risk signals—particularly for AD, BD, and depression—most simply explained by cis-regulatory effects on SLC41A1. This pattern indicates that SLC41A1 is not a disorder-specific locus but instead functions as a transdiagnostic vulnerability factor influencing key cellular processes, such as magnesium homeostasis, which consequently affects neuronal excitability and synaptic plasticity [12,13,14,37,38,39]. Unlike earlier observational studies linking magnesium imbalance or SLC41A1 variants to individual disorders without establishing causality [40,41], our integrated MR-colocalization approach provides more definitive, causally grounded evidence that SLC41A1 acts as a risk-modifying factor in neuropsychiatric conditions. These results offer a framework for investigating how SLC41A1-dependent magnesium signaling contributes to disease-relevant changes in gene expression, synaptic function, and neural circuitry.

At the transcriptomic level, our findings indicate that SLC41A1 contributes to neuropsychiatric risk in a disease- and state-dependent manner, rather than through a uniform mechanism across disorders. SLC41A1 expression was markedly elevated in AD and BD, whereas no significant differential expression was observed in depression, despite robust genetic associations from MR analyses. This variability may reflect the gene’s involvement in stable cellular processes—such as ionic regulation, mitochondrial function, and synaptic vesicle dynamics—rather than acute disease states. Conversely, the absence of expression changes in depression suggests that SLC41A1 regulation in this context may be subtler, region-specific, or dynamically linked to stress-related states, which are challenging to capture in bulk transcriptomic datasets.

Functionally, SLC41A1 appears to regulate key pathways associated with magnesium homeostasis, synaptic plasticity, and bioenergetic metabolism. Enrichment analyses of SLC41A1-associated DEGs revealed significant overrepresentation of pathways related to magnesium transport, intracellular magnesium regulation, synaptic vesicle dynamics, and mitochondrial function, consistent with its established role as a magnesium transporter [15,16,20,44,45]. Notably, the enrichment of glutamatergic and other synaptic pathways suggests that SLC41A1 may influence neuropsychiatric risk by modulating glutamate receptor-mediated plasticity. Glutamatergic dysregulation has been widely implicated in several neuropsychiatric disorders [46,47,48], although the specific contribution of SLC41A1 to these mechanisms remains to be elucidated.

Network-level analyses provide further insight into the molecular role of SLC41A1 in NMDA receptor-mediated plasticity. Our results revealed a strong connection between SLC41A1 and NMDA receptor-related genes (e.g., *GRINA*, *CAMK2G* and *GRIN2C*), especially in brain regions such as the amygdala and hippocampus. *GRINA*, an auxiliary protein of the NMDA receptor subunit, is known to modulate receptor function and interact with other components of the glutamatergic system. While prior studies have examined the roles of *GRINA*, *GRIN2C*, and *GRIN3B* in NMDA receptor signaling and neuropsychiatric disorders [42,43,44], our study provides the first evidence connecting SLC41A1 to this molecular network. Furthermore, the amygdala and hippocampus—regions integral to emotional regulation and memory—exhibit the strongest associations with SLC41A1, suggesting region-specific effects of SLC41A1 on NMDA receptor function. These findings highlight SLC41A1 as a potential therapeutic target in disorders such as BD and AD, where NMDA receptor dysfunction is well established [45,46].

In the present study, electrophysiological recordings from the Schaffer collateral–CA1 pathway showed that pharmacological perturbation with imipramine reduced NMDAR-mediated fEPSP amplitudes. However, imipramine is not a selective SLC41A1 inhibitor and may exert off-target effects in hippocampal slice preparations, including actions on monoaminergic systems, receptors, and ion channels. Therefore, the pharmacological data alone cannot establish SLC41A1 specificity. To address this limitation, we performed AAV-mediated SLC41A1 shRNA knockdown in the hippocampal CA1 region and observed a similar reduction in NMDAR-mediated fEPSP amplitudes compared with scramble controls. Together, these convergent pharmacological and genetic results support the involvement of SLC41A1 in regulating NMDA receptor-mediated synaptic responses, with the shRNA experiment providing the more specific mechanistic evidence. Although the precise biophysical mechanisms remain unclear, SLC41A1-mediated magnesium transport probably alters the intracellular Mg^2+^ environment, thereby modulating NMDA receptor function and synaptic plasticity [49,50]. This model aligns with existing evidence connecting NMDA receptor dysfunction to mood and cognitive disorders and with the rapid antidepressant effects of NMDA antagonists [51,52,53,54]. Collectively, these findings suggest that SLC41A1 serves as an upstream regulator of NMDA receptor function, offering a promising therapeutic entry point for targeting magnesium-dependent glutamatergic signaling in neuropsychiatric disorders.

Taken together, these findings position SLC41A1 as a magnesium-centered regulatory node within a shared pathophysiological axis across mood and cognitive disorders, rather than as a disorder-specific marker. Prior research linking magnesium imbalance to neuropsychiatric disorders has largely relied on observational studies of peripheral magnesium levels or single-disease cohorts, limiting the ability to distinguish causality from correlation and to integrate magnesium-related findings into a coherent mechanistic model. Although isolated reports have implicated individual magnesium transporters or synaptic pathways in specific disorders, their cross-disorder relevance and integration with downstream glutamatergic mechanisms have remained unexplored.

In contrast, the present study combines MR, colocalization, patient-derived transcriptomics, network analyses, and in vitro electrophysiology to converge on SLC41A1 as a transdiagnostic modifier linking genetically determined variation in magnesium regulation to NMDA receptor-dependent synaptic plasticity. This integrated framework minimizes confounding factors at the genetic level and provides direct experimental evidence for how SLC41A1 activity may influence circuit-level function. In doing so, it advances the field from descriptive associations between magnesium status and neuropsychiatric outcomes toward a testable mechanistic model centered on a specific magnesium transporter and its downstream effects on glutamatergic signaling.

Several limitations should be acknowledged. First, the transcriptomic and co-expression analyses are based on bulk tissue data, which cannot resolve cell-type-specific or dynamic patterns of SLC41A1 regulation. Future applications of single-cell or spatial transcriptomics will be essential to elucidate the cellular heterogeneity of SLC41A1 function in the human brain. Second, the genetic instruments for SLC41A1 expression were derived from whole-blood eQTLs rather than brain-specific datasets, creating a potential tissue–context mismatch. However, the eQTLGen consortium provides substantially higher statistical power than current brain-specific resources, and prior research has established a high genetic correlation (r_b_ = 0.70) for cis-eQTL effects between blood and brain [55]. This cross-tissue proxy is further supported by our independent brain transcriptomic validation and, more crucially, by our functional experiments using a selective SLC41A1 inhibitor in hippocampal slices. Third, although pathway enrichment and co-expression networks suggest that SLC41A1 is involved in magnesium, mitochondrial, and synaptic processes, these approaches cannot establish causal relationships. Further investigations using cellular and circuit-level models are needed to define how SLC41A1 and its interacting partners modulate these pathways. Fourth, the experimental validation was limited to a single in vitro hippocampal slice model. While the use of a selective SLC41A1 inhibitor confirmed the functional link to NMDA receptors, extending these studies to additional brain regions, developmental stages, and in vivo systems will be crucial for clarifying the broader physiological and pathological relevance of SLC41A1-NMDA coupling.

## 5. Conclusions

In summary, this multi-level investigation identifies SLC41A1 as a transdiagnostic modifier of neuropsychiatric risk and establishes a mechanistic link between magnesium-dependent cellular homeostasis and NMDA receptor-mediated synaptic plasticity. Through the integration of MR and colocalization analyses with patient-derived transcriptomics, network modeling, and electrophysiology, we provide convergent evidence that genetically increased SLC41A1 expression perturbs magnesium-sensitive glutamatergic signaling within stress-responsive neural circuits. These findings position SLC41A1 as a potential upstream regulator of mood- and cognition-related network dysfunction and as a promising axis for biomarker discovery and therapeutic intervention. Future work employing higher-resolution, human-derived cellular and circuit models will be critical to further elucidate its role across the neuropsychiatric spectrum.

## Figures and Tables

**Table 1:** Assessment of heterogeneity and horizontal pleiotropy in the Mendelian randomization analysis of SLC41A1 expression across thirteen neuropsychiatric disorders.

Outcomes	Methods	Cochran’s Q	df	*p* for Cochran’s Q	MR-Egger Intercept	*p* for MR- Egger Intercept	*p* for MR-PRESSO Global
Alzheimer’s Disease	MR-Egger	9.387	8	0.311	−0.0038	0.811	0.518
	Inverse Variance Weighted	9.459	9	0.396			
Parkinson’s Disease	MR-Egger	14.872	8	0.062	−0.0283	0.332	0.095
	Inverse Variance Weighted	16.855	9	0.051			
Post-Traumatic Stress Disorder	MR-Egger	6.126	8	0.633	0.0532	0.078	0.412
	Inverse Variance Weighted	10.210	9	0.334			
Bipolar Disorder	MR-Egger	3.870	5	0.568	0.0024	0.828	0.730
	Inverse Variance Weighted	3.923	6	0.687			
Autism Spectrum Disorder	MR-Egger	4.692	6	0.584	0.0130	0.449	0.637
	Inverse Variance Weighted	5.347	7	0.618			
Alcohol Dependence	MR-Egger	2.849	8	0.943	0.0012	0.936	0.950
	Inverse Variance Weighted	2.856	9	0.970			
Obsessive–Compulsive Disorder	MR-Egger	8.766	14	0.846	−0.0285	0.359	0.880
	Inverse Variance Weighted	9.664	15	0.840			
Attention Deficit Hyperactivity Disorder	MR-Egger	3.150	5	0.677	−0.0271	0.229	0.554
	Inverse Variance Weighted	5.031	6	0.540			
Schizophrenia	MR-Egger	6.944	15	0.959	−0.0069	0.462	0.964
	Inverse Variance Weighted	7.513	16	0.962			
Anxiety Disorder	MR-Egger	9.017	15	0.877	0.0296	0.220	0.793
	Inverse Variance Weighted	10.653	16	0.830			
Depression	MR-Egger	5.158	8	0.741	0.0105	0.024	0.306
	Inverse Variance Weighted	12.859	9	0.169			
Anorexia Nervosa	MR-Egger	1.775	3	0.620	−0.0003	0.994	0.845
	Inverse Variance Weighted	1.775	4	0.777			
Smoking	MR-Egger	10.866	8	0.209	0.0051	0.262	0.230
	Inverse Variance Weighted	12.844	9	0.170			

Cochran’s Q: test for heterogeneity across instrumental variables. MR-Egger intercept: test for horizontal pleiotropy. MR-PRESSO global: global test for horizontal pleiotropy and outlier effects. *p* < 0.05 indicates statistical significance.

## Data Availability

The datasets used in this study are available from public online repositories. Access details and accession numbers can be found in the article or Supplementary Materials. Animal data are available upon reasonable request from the corresponding author.

## Supplementary Materials

The following supporting information can be downloaded at: https://www.mdpi.com/article/10.3390/biomedicines14030610/s1, Supplemental Methods: Supplemental Text S1: Regulatory network analysis of SLC41A1; Supplemental Results: Supplemental Table S1: Characteristics of SNPs for SLC41A1 expression used as instruments in Mendelian randomization analysis; Supplemental Table S2: Summary of Mendelian randomization analysis for SLC41A1 expression and thirteen neuropsychiatric disorders; Supplemental Table S3: Posterior Probability of colocalization (PPH0–PPH4) for SLC41A1 expression and neuropsychiatric disorders; Supplemental Table S4: Gene Ontology (GO) Enrichment Analysis of SLC41A1 Gene and Its Enrichment in Various GO Terms; Supplemental Figure S1: Leave-one-out analyses for thirteen neuropsychiatric outcomes; Supplemental Figure S2: Regulatory Network of SLC41A1 Involving miRNAs and Transcription Factors; Supplemental Figure S3: The full western blot images of SLC41A1 shRNA Knockdown efficiency.

## Abbreviations

The following abbreviations are used in this manuscript:

AD	Alzheimer’s Disease
PD	Parkinson’s Disease
BD	Bipolar Disorder
NMDA	N-Methyl-D-Aspartate
SLC41A1	Solute Carrier Family 41 Member 1
SNP	Single-Nucleotide Polymorphisms
MR	Mendelian Randomization
eQTL	Expression Quantitative Trait Loci
GWAS	Genome-Wide Association Study
PTSD	Posttraumatic Stress Disorder
OCD	Obsessive–Compulsive Disorder
ADHD	Attention-Deficit Hyperactivity Disorder
ASD	Autism Spectrum Disorder
GEO	Gene Expression Omnibus
IVW	Inverse variance Weighted
PPHs	Posterior Probability of Hypothesis
DEGs	Differentially Expressed Genes
GO	Gene Ontology
KEGG	Kyoto Encyclopedia of Genes and Genomes
ssGSEA	Single-sample Gene Set Enrichment Analysis
FDR	False Discovery Rate
fEPSPs	Field Excitatory Postsynaptic Potentials
