# Supplementary material for: Magnesium Transporter SLC41A1 Links Magnesium Homeostasis to NMDA Receptor-Related Synaptic Dysfunction: A Transdiagnostic Therapeutic Target for Neuropsychiatric Disorders
Source: Biomedicines. 2026 Mar 9;14(3):610. doi: 10.3390/biomedicines14030610 (PMC13024283; doi:10.3390/biomedicines14030610)
Supplement: Supplementary file 1 [file biomedicines-14-00610-s001.zip › biomedicines-4070533-supplementary.pdf]

Supplementary Material

Magnesium Transporter SLC41A1 Links Magnesium Homeostasis to NMDA Receptor-Related Synaptic Dysfunction: A Transdiagnostic Therapeutic Target for Neuropsychiatric Disorders

Supplemental Methods

Supplemental Text S1 Regulatory network analysis of SLC41A1 2

Supplemental Results

Supplemental Table S1 Characteristic of instrumental variables (IV) in Mendelian randomization analysis 3
Supplemental Table S2 Mendelian randomization analysis for all outcomes with five estimators 4
Supplemental Table S3 Posterior Probability of colocalization 8
Supplemental Table S4 Gene Ontology (GO) Enrichment Analysis 9
Supplemental Figure S1 Leave-one-out analyses 10
Supplemental Figure S2 Regulatory Network of SLC41A1 12
Supplemental Figure S3. The full western blot images of SLC41A1 shRNA Knockdown efficiency 13

Supplemental Methods

Supplemental Text S1. Regulatory network analysis of SLC41A1

To investigate the upstream regulators of SLC41A1, miRNAs and transcription factors (TFs) were identified using data from miRTarBase and ENCODE, respectively. miRTarBase, a database of experimentally validated miRNA-target interactions, was used to retrieve miRNAs that regulate SLC41A1, while TFs binding to SLC41A1 were identified from ENCODE data. These interactions were then used to construct the miRNA-SLC41A1 and TF-SLC41A1 regulatory networks. The networks were visualized using 'Cytoscape' v3.9.0, and topological metrics, including degree centrality and betweenness centrality, were calculated to identify hub regulators with the highest connectivity and influence within the network.

Supplemental Results

Supplementary Table S1. Characteristics of SNPs for SLC41A1 expression used as instruments in Mendelian randomization analysis

SNP	Effect Allele	Other Allele	Beta	SE	EAF	*p* value	MAF	F-statistic
rs823075	T	C	0.21453	0.00803926	0.398298	6.97E-157	0.398298	712.1048054
rs77358687	T	G	-0.180228	0.00861863	0.507266	4.22E-97	0.492734	437.288497
rs1775140	A	C	0.138228	0.0101608	0.423541	3.79E-42	0.423541	185.0700927
rs112215351	C	T	-0.113479	0.0106085	0.207613	1.05E-26	0.207613	114.4255579
rs140787532	G	A	-0.110866	0.0107001	0.316371	3.72E-25	0.316371	107.3547054
rs189456446	A	G	-0.10316	0.0105468	0.398928	1.36E-22	0.398928	95.67120541
rs12759896	T	C	-0.105317	0.0150643	0.253363	2.73E-12	0.253363	48.87638164
rs12081859	A	G	0.0580814	0.00954878	0.223428	1.18E-09	0.223428	36.998011
rs11240545	T	G	0.0776059	0.0095694	0.353378	5.07E-16	0.353378	65.76882194
rs12735654	A	C	0.0646949	0.0105586	0.220979	8.94E-10	0.220979	37.54286544
rs7528211	A	G	-0.0777224	0.0116876	0.183671	2.93E-11	0.183671	44.22234069
rs79809641	A	G	0.121066	0.025591	0.0338029	2.24E-06	0.0338029	22.38050458
rs9438391	G	A	-0.0814072	0.012763	0.179173	1.79E-10	0.179173	40.68366752
rs77611692	T	A	0.111723	0.0232592	0.0411662	1.56E-06	0.0411662	23.0725519
rs11240525	C	A	-0.0546279	0.0108756	0.162419	5.09E-07	0.162419	25.23030842
rs61824663	A	G	0.128505	0.0229514	0.0344916	2.16E-08	0.0344916	31.34885535
rs112374673	A	G	-0.111391	0.0166236	0.0656023	2.07E-11	0.0656023	44.90038295
rs58086427	A	G	-0.0909658	0.0181786	0.111512	5.62E-07	0.111512	25.04006313
rs28894813	G	A	-0.130289	0.0199481	0.0828584	6.52E-11	0.0828584	42.65917264
rs149872882	A	G	-0.185067	0.0281985	0.0252113	5.27E-11	0.0252113	43.07308281
rs150855094	A	C	0.432764	0.0868794	0.0177719	6.32E-07	0.0177719	24.81239159
rs149560808	G	A	0.423867	0.0878953	0.0182198	1.42E-06	0.0182198	23.25561915
rs61814947	G	A	0.805347	0.14559	0.0133979	3.17E-08	0.0133979	30.5987016
rs72748980	A	G	-0.0897075	0.0170956	0.062779	1.54E-07	0.062779	27.53523657

SNP, Single Nucleotide Polymorphism; SE, Standard Error; EAF, Effect Allele Frequency; MAF, Minor Allele Frequency.

Supplementary Table S2. Summary of Mendelian randomization analysis for SLC41A1 expression and thirteen neuropsychiatric disorders

Outcome	Method	nSNP	Beta	SE	p value	Lower CI	Upper CI	OR	OR_95% CI_lower	OR_95% CI_upper
Alzheimer's Disease	MR Egger	10	0.2072	0.1158	0.1113	-0.0197	0.4342	1.2303	0.9805	1.5438
Alzheimer's Disease	Weighted median	10	0.2084	0.0553	0.0002	0.1001	0.3167	1.2317	1.1053	1.3726
Alzheimer's Disease	Inverse variance weighted	10	0.1814	0.0475	0.0001	0.0883	0.2745	1.1989	1.0924	1.3158
Alzheimer's Disease	Simple mode	10	0.1582	0.0843	0.0933	-0.0070	0.3234	1.1714	0.9930	1.3818
Alzheimer's Disease	Weighted mode	10	0.2050	0.0564	0.0054	0.0946	0.3155	1.2276	1.0992	1.3710
Parkinson's Disease	MR Egger	10	0.3913	0.2066	0.0948	-0.0136	0.7961	1.4789	0.9865	2.2170
Parkinson's Disease	Weighted median	10	0.2487	0.0813	0.0022	0.0892	0.4081	1.2823	1.0933	1.5039
Parkinson's Disease	Inverse variance weighted	10	0.1990	0.0898	0.0267	0.0230	0.3750	1.2202	1.0233	1.4551
Parkinson's Disease	Simple mode	10	-0.1978	0.2802	0.4982	-0.7470	0.3514	0.8206	0.4738	1.4211
Parkinson's Disease	Weighted mode	10	0.2736	0.0853	0.0107	0.1064	0.4408	1.3147	1.1123	1.5540
Post-traumatic stress disorder	MR Egger	10	-0.1750	0.1991	0.4049	-0.5652	0.2151	0.8394	0.5683	1.2400
Post-traumatic stress disorder	Weighted median	10	0.1882	0.1079	0.0812	-0.0233	0.3998	1.2071	0.9769	1.4915
Post-traumatic stress disorder	Inverse variance weighted	10	0.1878	0.0916	0.0402	0.0084	0.3673	1.2066	1.0084	1.4438
Post-traumatic stress disorder	Simple mode	10	0.4589	0.2142	0.0608	0.0391	0.8787	1.5823	1.0399	2.4077
Post-traumatic stress disorder	Weighted mode	10	0.1621	0.1098	0.1739	-0.0531	0.3772	1.1759	0.9483	1.4582
Bipolar disorder	MR Egger	7	0.1135	0.0788	0.2095	-0.0410	0.2679	1.1201	0.9598	1.3072
Bipolar disorder	Weighted median	7	0.1421	0.0418	0.0007	0.0601	0.2240	1.1527	1.0620	1.2511
Bipolar disorder	Inverse variance weighted	7	0.1293	0.0375	0.0006	0.0559	0.2028	1.1381	1.0575	1.2248
Bipolar disorder	Simple mode	7	0.1459	0.0742	0.0970	0.0004	0.2914	1.1570	1.0004	1.3383
Bipolar disorder	Weighted mode	7	0.1428	0.0446	0.0185	0.0555	0.2302	1.1535	1.0570	1.2588
Autism Spectrum Disorder	MR Egger	8	0.0390	0.1161	0.7482	-0.1886	0.2667	1.0398	0.8281	1.3056
Autism Spectrum Disorder	Weighted median	8	0.1064	0.0552	0.0538	-0.0017	0.2145	1.1123	0.9983	1.2392

Outcome	Method	nSNP	Beta	SE	p value	Lower CI	Upper CI	OR	OR_95% CI_lower	OR_95% CI_upper
Autism Spectrum Disorder	Inverse variance weighted	8	0.1248	0.0475	0.0086	0.0317	0.2179	1.1330	1.0322	1.2435
Autism Spectrum Disorder	Simple mode	8	0.1321	0.0905	0.1877	-0.0453	0.3095	1.1412	0.9557	1.3627
Autism Spectrum Disorder	Weighted mode	8	0.0975	0.0563	0.1269	-0.0128	0.2077	1.1024	0.9873	1.2309
Alcohol dependence	MR Egger	10	0.1553	0.1041	0.1741	-0.0487	0.3592	1.1680	0.9525	1.4323
Alcohol dependence	Weighted median	10	0.1454	0.0564	0.0099	0.0350	0.2559	1.1565	1.0356	1.2916
Alcohol dependence	Inverse variance weighted	10	0.1631	0.0450	0.0003	0.0749	0.2513	1.1772	1.0778	1.2857
Alcohol dependence	Simple mode	10	0.1799	0.0888	0.0734	0.0059	0.3540	1.1971	1.0059	1.4247
Alcohol dependence	Weighted mode	10	0.1272	0.0575	0.0544	0.0144	0.2399	1.1356	1.0145	1.2711
Obsessive-compulsive disorder	MR Egger	16	0.3314	0.2329	0.1766	-0.1250	0.7878	1.3929	0.8825	2.1986
Obsessive-compulsive disorder	Weighted median	16	0.1675	0.1131	0.1387	-0.0542	0.3891	1.1823	0.9472	1.4757
Obsessive-compulsive disorder	Inverse variance weighted	16	0.1272	0.0883	0.1495	-0.0458	0.3002	1.1357	0.9553	1.3501
Obsessive-compulsive disorder	Simple mode	16	0.2100	0.1655	0.2239	-0.1144	0.5345	1.2337	0.8919	1.7066
Obsessive-compulsive disorder	Weighted mode	16	0.2043	0.1203	0.1100	-0.0314	0.4400	1.2267	0.9691	1.5527
Attention Deficit Hyperactivity Disorder	MR Egger	7	0.3320	0.1430	0.0680	0.0516	0.6123	1.3937	1.0530	1.8447
Attention Deficit Hyperactivity Disorder	Weighted median	7	0.1946	0.0671	0.0037	0.0632	0.3261	1.2149	1.0652	1.3855
Attention Deficit Hyperactivity Disorder	Inverse variance weighted	7	0.1531	0.0587	0.0091	0.0380	0.2682	1.1654	1.0387	1.3076
Attention Deficit Hyperactivity Disorder	Simple mode	7	-0.0839	0.1466	0.5881	-0.3713	0.2035	0.9196	0.6899	1.2257
Attention Deficit Hyperactivity Disorder	Weighted mode	7	0.2108	0.0699	0.0235	0.0738	0.3478	1.2347	1.0766	1.4160
Schizophrenia	MR Egger	17	0.1046	0.0730	0.1726	-0.0385	0.2478	1.1103	0.9622	1.2812

Outcome	Method	nSNP	Beta	SE	p value	Lower CI	Upper CI	OR	OR_95% CI_lower	OR_95% CI_upper
Schizophrenia	Weighted median	17	0.0592	0.0348	0.0889	-0.0090	0.1274	1.0610	0.9910	1.1359
Schizophrenia	Inverse variance weighted	17	0.0534	0.0270	0.0482	0.0004	0.1064	1.0549	1.0004	1.1123
Schizophrenia	Simple mode	17	0.0552	0.0524	0.3077	-0.0475	0.1579	1.0567	0.9536	1.1710
Schizophrenia	Weighted mode	17	0.0552	0.0397	0.1837	-0.0227	0.1330	1.0567	0.9776	1.1423
Anxiety disorder	MR Egger	17	-0.0565	0.1941	0.7748	-0.4370	0.3239	0.9450	0.6460	1.3826
Anxiety disorder	Weighted median	17	0.1447	0.1081	0.1806	-0.0671	0.3565	1.1557	0.9351	1.4284
Anxiety disorder	Inverse variance weighted	17	0.1698	0.0799	0.0337	0.0131	0.3264	1.1850	1.0132	1.3860
Anxiety disorder	Simple mode	17	0.2842	0.1607	0.0960	-0.0307	0.5992	1.3287	0.9697	1.8206
Anxiety disorder	Weighted mode	17	0.1398	0.1146	0.2399	-0.0847	0.3644	1.1501	0.9188	1.4396
Depression	MR Egger	10	0.0247	0.0333	0.4797	-0.0406	0.0899	1.0250	0.9602	1.0941
Depression	Weighted median	10	0.0746	0.0194	0.0001	0.0366	0.1127	1.0775	1.0373	1.1193
Depression	Inverse variance weighted	10	0.1051	0.0196	0.0000	0.0668	0.1435	1.1109	1.0691	1.1543
Depression	Simple mode	10	0.1351	0.0458	0.0162	0.0453	0.2248	1.1446	1.0464	1.2521
Depression	Weighted mode	10	0.0787	0.0194	0.0029	0.0407	0.1168	1.0819	1.0415	1.1239
Anorexia nervosa	MR Egger	5	-0.1877	0.2448	0.4990	-0.6676	0.2922	0.8288	0.5129	1.3393
Anorexia nervosa	Weighted median	5	-0.1942	0.1204	0.1067	-0.4302	0.0417	0.8235	0.6504	1.0426
Anorexia nervosa	Inverse variance weighted	5	-0.1896	0.1112	0.0881	-0.4076	0.0283	0.8273	0.6653	1.0287
Anorexia nervosa	Simple mode	5	-0.2368	0.1984	0.2985	-0.6257	0.1520	0.7891	0.5349	1.1641
Anorexia nervosa	Weighted mode	5	-0.1924	0.1269	0.2042	-0.4411	0.0564	0.8250	0.6433	1.0580
Smoking	MR Egger	10	-0.0015	0.0218	0.9475	-0.0443	0.0413	0.9985	0.9567	1.0422
Smoking	Weighted median	10	0.0293	0.0149	0.0492	0.0001	0.0585	1.0297	1.0001	1.0602
Smoking	Inverse variance weighted	10	0.0199	0.0130	0.1259	-0.0056	0.0455	1.0201	0.9944	1.0465

Outcome	Method	nSNP	Beta	SE	p value	Lower CI	Upper CI	OR	OR_95% CI_lower	OR_95% CI_upper
Smoking	Simple mode	10	0.0490	0.0249	0.0805	0.0002	0.0977	1.0502	1.0002	1.1026
Smoking	Weighted mode	10	0.0344	0.0162	0.0627	0.0026	0.0661	1.0350	1.0026	1.0684

SNP, Single Nucleotide Polymorphism; SE, Standard Error; .CI, Confidence Intervals; OR, Odds Ratio.

Supplementary Table S3. Posterior Probability of colocalization (PPH0–PPH4) for SLC41A1 expression and neuropsychiatric disorders

**Outcome**	**PPH0**	**PPH1**	**PPH2**	**PPH3**	**PPH4**
Alzheimer's Disease	8.48E-151	0.113678265	4.33E-152	0.005790243	0.880531492
Parkinson's Disease	2.81E-150	0.376719448	7.47E-152	0.010001449	0.613279103
Post-traumatic stress disorder	6.58E-150	0.882141336	1.25E-151	0.016766286	0.101092379
Bipolar disorder	4.07E-151	0.054587388	3.99E-152	0.005329058	0.940083554
Autism Spectrum Disorder	4.99E-150	0.669057749	1.85E-151	0.024742866	0.306199385
Alcohol dependence	5.20E-150	0.697242938	9.13E-152	0.012228006	0.290529055
Attention Deficit Hyperactivity Disorder	5.41E-150	0.724669235	2.51E-151	0.033570352	0.241760413
Schizophrenia	5.72E-150	0.767298033	2.23E-151	0.029818191	0.202883775
Anxiety disorder	5.51E-150	0.738490557	1.36E-151	0.018246342	0.243263102
Depression	1.41E-151	0.018879689	5.55E-152	0.007417139	0.973703172

PPH, Posterior Probability of Colocalization.

Supplementary Table S4. Gene Ontology (GO) Enrichment Analysis of SLC41A1 Gene and Its Enrichment in Various GO Terms

ID	ONTOLOGY	Description	GeneRatio	*p* value	Adjust *p* value	Q value	Count
GO:0061768	MF	magnesium:sodium antiporter activity	2/18496	0.00011	0.00097	NA	1
GO:0010961	BP	intracellular magnesium ion homeostasis	4/18870	0.00021	0.00185	NA	1
GO:0071286	BP	cellular response to magnesium ion	7/18870	0.00037	0.00185	NA	1
GO:0010960	BP	magnesium ion homeostasis	11/18870	0.00058	0.00185	NA	1
GO:1903830	BP	magnesium ion transmembrane transport	17/18870	0.00090	0.00185	NA	1
GO:0015693	BP	magnesium ion transport	18/18870	0.00095	0.00185	NA	1
GO:0032026	BP	response to magnesium ion	19/18870	0.00101	0.00185	NA	1
GO:0015095	MF	magnesium ion transmembrane transporter activity	16/18496	0.00087	0.00389	NA	1
GO:0140828	MF	metal cation:monoatomic cation antiporter activity	41/18496	0.00222	0.00665	NA	1
GO:0035725	BP	sodium ion transmembrane transport	180/18870	0.00954	0.01446	NA	1
GO:0071248	BP	cellular response to metal ion	200/18870	0.01060	0.01446	NA	1
GO:0071241	BP	cellular response to inorganic substance	229/18870	0.01214	0.01446	NA	1
GO:0006814	BP	sodium ion transport	248/18870	0.01314	0.01446	NA	1
GO:0016323	CC	basolateral plasma membrane	249/19886	0.01252	0.01514	NA	1
GO:0009925	CC	basal plasma membrane	282/19886	0.01418	0.01514	NA	1
GO:0045178	CC	basal part of cell	301/19886	0.01514	0.01514	NA	1
GO:0015297	MF	antiporter activity	135/18496	0.00730	0.01528	NA	1
GO:0015081	MF	sodium ion transmembrane transporter activity	157/18496	0.00849	0.01528	NA	1
GO:0010038	BP	response to metal ion	359/18870	0.01902	0.01902	NA	1
GO:0022853	MF	active monoatomic ion transmembrane transporter activity	244/18496	0.01319	0.01979	NA	1
GO:0015291	MF	secondary active transmembrane transporter activity	286/18496	0.01546	0.01988	NA	1
GO:0046873	MF	metal ion transmembrane transporter activity	441/18496	0.02384	0.02455	NA	1
GO:0022804	MF	active transmembrane transporter activity	454/18496	0.02455	0.02455	NA	1

Supplemental Figure S1. Leave-one-out analyses for thirteen neuropsychiatric outcomes.

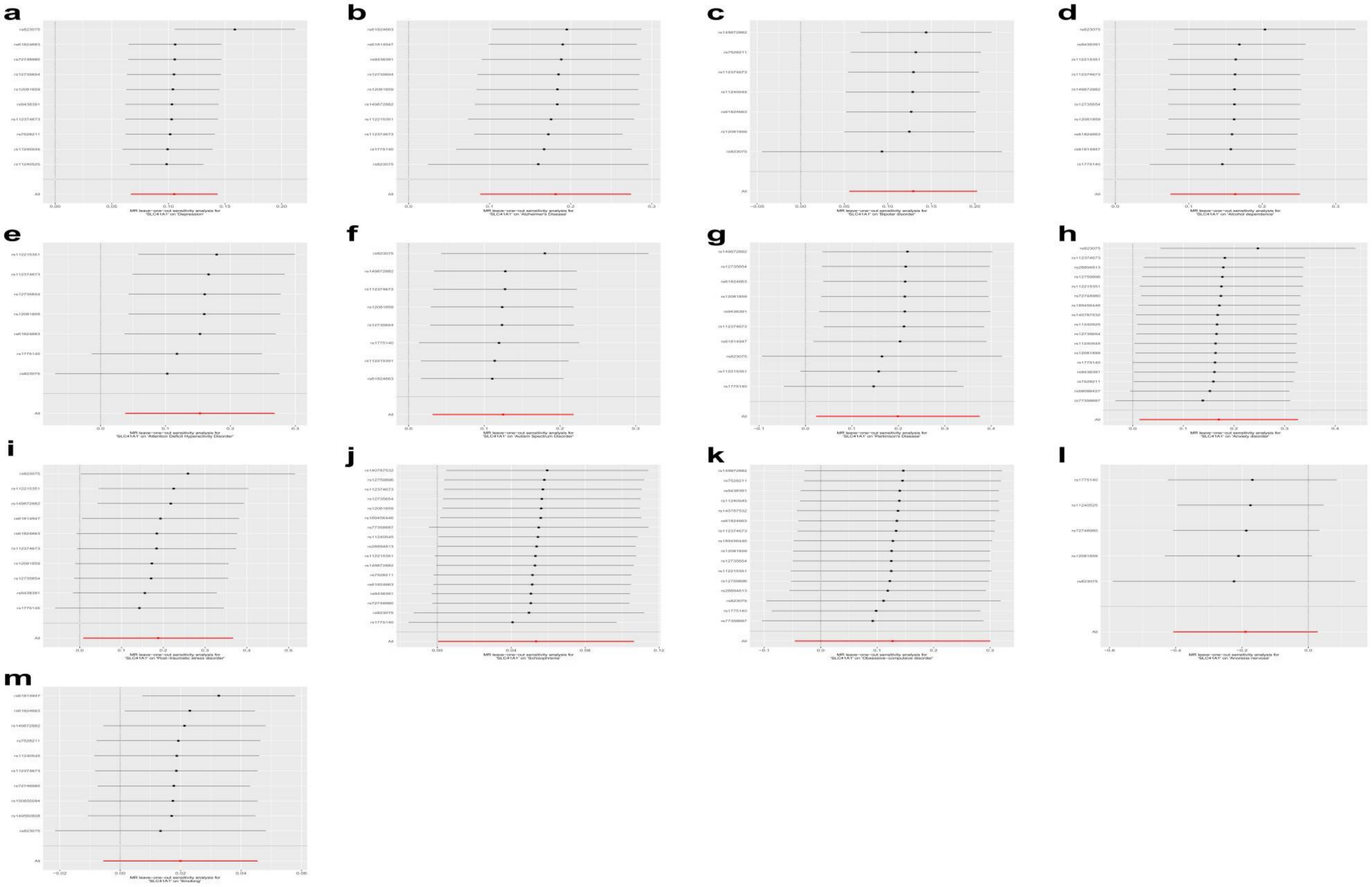

Leave-one-out sensitivity analyses were conducted for each outcome by sequentially excluding individual SNPs and re-estimating the causal effect of SLC41A1 expression. Forest plots display the causal estimates (odds ratios with 95% confidence intervals) recalculated after removing one SNP at a time. The vertical dashed line indicates the overall IVW estimate derived from all instruments. No single SNP was found to disproportionately influence the causal estimates across any outcome.

Panels correspond to the following outcomes: (a) Depression, (b) Alzheimer's disease, (c) Bipolar disorder, (d) Alcohol dependence, (e) Attention-deficit/hyperactivity disorder, (f) Autism spectrum disorder, (g) Parkinson's disease, (h) Anxiety disorder, (i) Post-traumatic stress disorder, (j) Schizophrenia, (k) Obsessive-compulsive disorder, (l) Anorexia nervosa, (m) Smoking behavior.

Supplemental Figure S2. Regulatory Network of SLC41A1 Involving miRNAs and Transcription Factors.

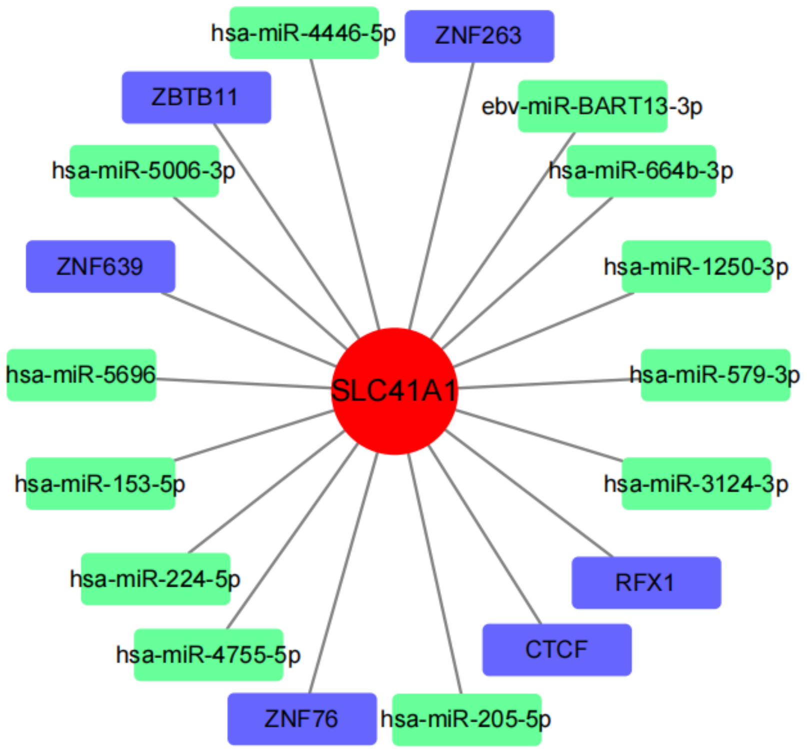

This figure illustrates the regulatory network of SLC41A1 involving 12 miRNAs and 6 transcription factors (TFs). The miRNAs are depicted in green, and the transcription factors are shown in blue. We identified potential miRNA and TFs of SLC41A1 using the miRTarBase and ENCODE databases. A total of 12 miRNAs were predicted to regulate SLC41A1, including hsa-miR-1250-3p, hsa-miR-4446-5p, and hsa-miR-4755-5p. These miRNAs were found to potentially regulate SLC41A1 through post-transcriptional mechanisms. Additionally, ENCODE data revealed that SLC41A1 interacts with several TFs, such as ZNF76,

ZBTB11, RFX1, ZNF639, ZNF263, and CTCF, which could play a role in the transcriptional regulation of SLC41A1. These findings were visualized in the miRNA-SLC41A1 and TF-SLC41A1 regulatory networks, highlighting the intricate regulation of SLC41A1 by both miRNAs and TFs.

Supplemental Figure S3. The full western blot images of SLC41A1 shRNA Knockdown efficiency.

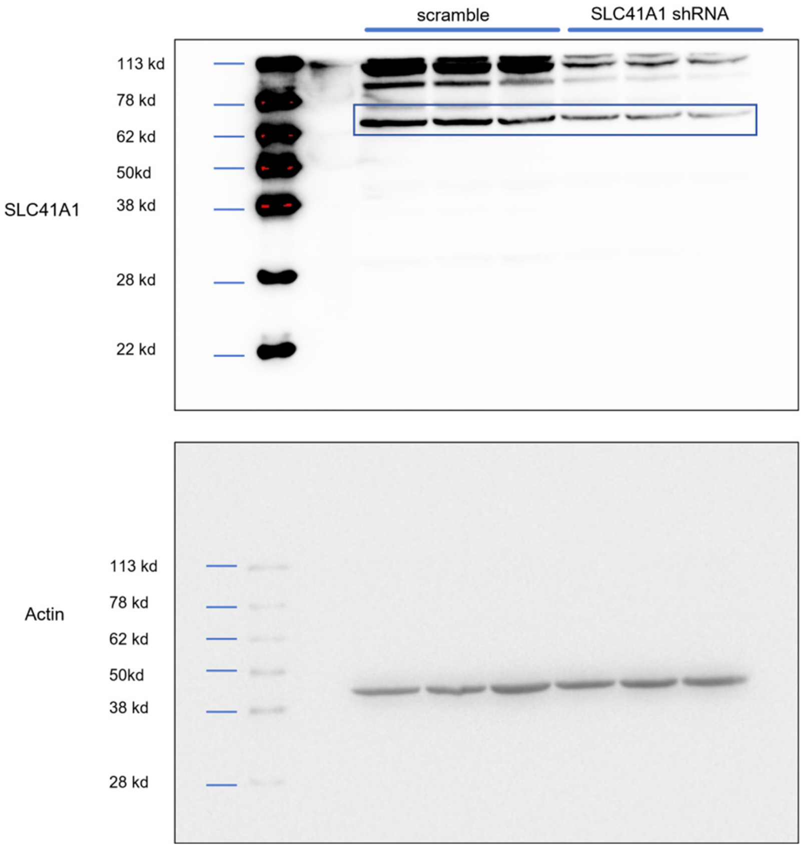

Total protein was extracted from the tissues using Tissue Protein Extraction Reagent (T-PER; Thermo Scientific, USA). Protein concentrations weredetermined

with a Pierce BCA Protein Assay Kit (Thermo Scientific, USA). After quantification, samples containing 20 μg of protein were separated by SDS-PAGE and transferred to PVDF membranes (Millipore, USA). The PVDF membranes were blocked with QuickBlock™ Blocking Buffer (Beyotime, China) and then incubated with primary antibodies overnight at 4℃. The primary antibodies were used as follows: SLC41A1 (1:1000, Invitrogen, USA), and beta-actin (1:1000, CST, USA). After extensive washing with 1×TBST (3 times), the membranes were incubated with HRP-conjugated secondary antibodies for 1 h at room temperature. Band optical intensities were digitized and quantified using ImageJ software, with normalization to beta-actin for accurate quantitative analysis. Following the detection of SLC41A1, the membrane was stripped to remove the primary and secondary antibodies. Subsequently, the membrane was re-probed with a primary antibody against actin (1:1000, CST, USA). All proteins were detected on the same blot.
